# On Bleeding from the Ear as a Consequence of Injury Done to the Chin

**Published:** 1857-12

**Authors:** 


*On Bleeding from the Ear as a Consequence of Injury done to the Chin.* By M. Morvan. (Archives Générales, cinquième série, tome viii, pp. 653–654.)

Bleeding from the ear as a consequence of *contre-coup* has been accepted by surgeons as an almost certain indication of fracture of the base of the cranium. M. Morvan has, however, met with two cases in which injury to the chin gave rise to this phenomenon. The subject of the first of these, was a robust lad, five years of age, who had, five or six hours before the author's arrival, fallen on his face on the pavement from a height of several feet. Immediately after the fall, a large flow of blood took place from the right ear, this being continued, when M. Morvan saw him, only in occasional drops, in which condition it lasted for three days longer. No fracture or dislocation of the jaw could be detected, and the membrana tympani was not ruptured. The child suffered much from pain in front of the right ear, from attempts at deglutition, and from any movement of the jaw.

In the second case, a very strong man, aged forty-seven, received a kick from a horse on the chin, which almost deprived him of consciousness, and gave rise to an abundant jet of blood from the right ear. No fracture or dislocation could be found, but deglutition was excessively difficult. Prompt depletion dissipated the cerebral symptoms, and all went on well, a considerable amount of deafness remaining in the right ear. The membrana tympani was uninjured.

On searching, the author has been able to find only three analogous cases, and these only meagrely detailed, making thus, with his own, five cases. In three of these the blow on the chin resulted from a fall, and in two was produced by a kick from a horse. In three, the bleeding took place from one ear, and in two from both ears. In the author's cases the force acted obliquely, and the bleeding occurred on the opposite side to that of the point of contact. When bleeding has occurred from both ears, the blow has been central. In one only of the five cases, did fracture of the jaw occur. In order to produce bleeding by this form of *contre-coup*, it is probably necessary that the shock should be entirely transmitted to the articulation of the jaw, while when fracture takes place, its force is usually exhausted in the production of the lesion of the bone.

In the three cases in which the point has been noted, the difficulty of deglutition and mastication has been excessive at first, and has continued for a long period; and M. Morvan suggests that the lesion which gives rise to this symptom, as well as the bleeding from the ear, is a fracture across the glenoid cavity, which explains the occurrence of the abundant hæmorrhage, the membrana tympani remaining entire. Some experiments he has made in the dead body, by inflicting blows upon the chin, have failed to produce this form of fracture, but have induced fracture of the base. Thus no doubt can exist that this description of *contre-coup* may also produce fracture of the base, with bleeding from the ear, and rupture of the membrana tympani, but when we meet with snch bleeding as a consequence of violence done to the chin, and without rupture of the membrane, the hæmorrhage may be regarded as a far less dangerous symptom.—*British and Foreign Medico-Chirurgical Review.*

